# Fiber-tip polymer clamped-beam probe for high-sensitivity nanoforce measurements

**DOI:** 10.1038/s41377-021-00611-9

**Published:** 2021-08-27

**Authors:** Mengqiang Zou, Changrui Liao, Shen Liu, Cong Xiong, Cong Zhao, Jinlai Zhao, Zongsong Gan, Yanping Chen, Kaiming Yang, Dan Liu, Ying Wang, Yiping Wang

**Affiliations:** 1 Key Laboratory of Optoelectronic Devices and Systems of Ministry of Education/GuangDong Province, College of Physics and Optoelectronic Engineering, Shenzhen University, Shenzhen, 518060 China; 2 Shenzhen Key Laboratory of Photonic Devices and Sensing Systems for Internet of Things, Guangdong and Hong Kong Joint Research Centre for Optical Fibre Sensors, Shenzhen University, Shenzhen, 518060 China; 3 College of Materials Science and Engineering, Shenzhen Key Laboratory of Polymer Science and Technology, Guangdong Research Center for Interfacial Engineering of Functional Materials, Shenzhen, 518060 China; 4 Wuhan National Laboratory for Optoelectronics (WNLO), Huazhong University of Science and Technology (HUST), Wuhan, 430074 China; 5 Shenzhen Huazhong University of Science and Technology Research Institute, Shenzhen, Guangdong, 518057 China

**Keywords:** Polymers, Optical sensors

## Abstract

Micromanipulation and biological, material science, and medical applications often require to control or measure the forces asserted on small objects. Here, we demonstrate for the first time the microprinting of a novel fiber-tip-polymer clamped-beam probe micro-force sensor for the examination of biological samples. The proposed sensor consists of two bases, a clamped beam, and a force-sensing probe, which were developed using a femtosecond-laser-induced two-photon polymerization (TPP) technique. Based on the finite element method (FEM), the static performance of the structure was simulated to provide the basis for the structural design. A miniature all-fiber micro-force sensor of this type exhibited an ultrahigh force sensitivity of 1.51 nm μN^−1^, a detection limit of 54.9 nN, and an unambiguous sensor measurement range of ~2.9 mN. The Young’s modulus of polydimethylsiloxane, a butterfly feeler, and human hair were successfully measured with the proposed sensor. To the best of our knowledge, this fiber sensor has the smallest force-detection limit in direct contact mode reported to date, comparable to that of an atomic force microscope (AFM). This approach opens new avenues towards the realization of small-footprint AFMs that could be easily adapted for use in outside specialized laboratories. As such, we believe that this device will be beneficial for high-precision biomedical and material science examination, and the proposed fabrication method provides a new route for the next generation of research on complex fiber-integrated polymer devices.

## Abstract (web summary)

Fiber-tip polymer clamped-beam probe for high-sensitivity nanoforce measurements are proposed for the first time. The proposed sensor has great application prospects for the mechanical properties examination of biological samples.

## Graphical abstract

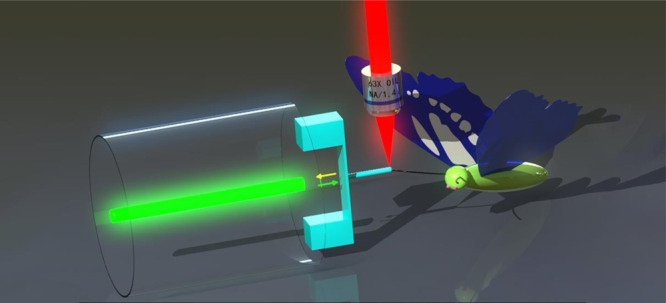

## Introduction

With the current trend of device miniaturization, micromanipulation has attracted considerable interest recently. In the micro world, it is necessary to detect and control the contact force reliably for the object is easy to be damaged^1^. Many fields, such as micro-systems^2^, biological sample examination^3^, microfluidic systems^4^, micro-assembly^5^, medicine^6^, and materials science^7^, require highly sensitive micro-force sensors. For instance, in medical cardiac catheterization, knowing the contact force between the catheter and the vascular wall is of great importance, which helps avoid damaging the patient’s fine vascular network. Over the past decade, a variety of micro-electro-mechanical system (MEMS) force sensors have been proposed, including capacitive-force sensors^8^, piezoelectric-type sensors^9^, and piezoresistive-force sensors^10^. However, MEMS force sensors are limited in many applications due to the need for proper packaging and poor electromagnetic compatibility^11^. Compared with MEMS force sensors, optical fiber ones have the advantages of high sensitivity, flexibility, lightweight, compactness, great biocompatibility, and immunity to electromagnetic interference^12–15^. Thus, a variety of fiber-optic force sensors have been reported during the past few years. In 2018, Shen et al.^16^ demonstrated a single-mode-fiber force sensor based on a tilted fiber Bragg grating (TFBG) for tension measuring of liquids, and this sensor achieved milli-Newton-level forces. In 2020, Donlagic et al. reported a thin silica diaphragm created at the optical fiber tip, forming a sealed Fabry–Perot interferometer (FPI) and achieved a force resolution of ~0.6 µN and a measurement range of ~0.6 mN^11^. However, due to its complex fabrication, the ultra-high Young’s modulus and stiffness limitation of the silica diaphragm leads to a low force resolution. Furthermore, the sealed FPI might be affected by the air pressure inside the closed cavity when detecting the external micro force.

Two-photon polymerization (TPP) lithography, which is induced by a femtosecond laser, with a resolution of less than 100 nm, is a three-dimensional (3D) microprinting technology. Applications have included metamaterials^17–19^, MEMS^20–22^, microfluidic devices^23–25^, and biomedicine^26–28^. Ideally, this TPP technology can be used to fabricate micro/nanometer structures of any shape. Even structures, such as micro-bionic or micro-magnetic driving mechanical structures that are hard to make through traditional processing, have been significantly developed with very good research prospects^29–32^. “Lab on fiber” technology, which equips the fiber with a variety of functions, is a concept about optical fiber processing and transformation. The optical fiber end face is an ideal platform to realize “lab on fiber”. Various new fiber-optical sensors, by using TPP technology on an optical fiber end face, have been manufactured and reported in recent years. Wang et al. reported a novel fiber-optical microphone based on polymerizing resonance grating waveguides on the end face of fiber for ultrasonic wave detection^33^. Melissinaki et al. proposed an optical fiber sensor utilizing femtosecond laser to directly write Fabry–Perot micro-optical sensor resonator on the fiber tip, which was used for common organic solvents detection^34^. We demonstrated a highly sensitive hydrogen sensor fabricated by using femtosecond laser directly polymerize micro-cantilever on fiber end face in our previous work^35^.

Depth-sensing indentation is the most commonly used technique that characterizes the mechanical properties of materials in micro/nanoscale. The most common instrument used for this indentation testing is an atomic force microscope (AFM)^36,37^. Despite its success, the instrument still has some drawbacks. The structure of AFM is complex and the volume of the AFM instrument is huge, which limits the AFM’s use in the environment outside the professional laboratory. In addition, when the sample is indented by AFM in liquid, it must be placed in a fluid chamber of only several square centimeters, in order to limit the size of the sample^38^. Furthermore, in the past few decades, the traditional AFM probes have remained basically unchanged in the aspects of material, structure, and processing method, hindering its application in the mechanical characterization of elastic-plastic materials seriously. To make probe-based micro-force sensors appropriate for micromanipulation and biological applications, it is necessary to reduce its size to a portable size so that they can adapt well when used outside the specialized labs, and flexible TPP microprinting methods (e.g., printing rounded probes) make it more convenient to test soft polymers, tissues, and cells.

In this work, we demonstrate the fabrication of a clamped-beam probe on a fiber tip by TPP 3D microprinting method, forming a highly integrated all-fiber micro-force sensor with an open-cavity FPI, which can be used to measure nano-Newton-level forces exerted by small objects. The structure of the sensor was optimized using the finite element method (FEM), and its static characteristic was analyzed. The force response of the sensor was measured to be 1.51 nm μN^−1^, which indicated two-orders-of-magnitude improvement over previously reported fiber-optic force sensor based on a modal interferometer (24.9 pm μN^−1^)^15^. The sensor exhibited an unambiguous measurement range of ~2.9 mN and an ultra-small detection limit of 54.9 nN, which is comparable to that achieved by AFMs. In addition, Young’s modulus of polydimethylsiloxane (PDMS), a butterfly feeler, and human hair were measured by the proposed sensor, and the accuracy of the results was verified using an AFM. This all-fiber-polymer force sensor exhibited high force sensitivity, an ultra-small detection limit, biocompatibility, good stability, and ultra-compactness. As such, a force-sensing element of this type would be a beneficial tool for biomechanics and material science measurements.

## Results

### Sensor construction

**Fig. 1:**
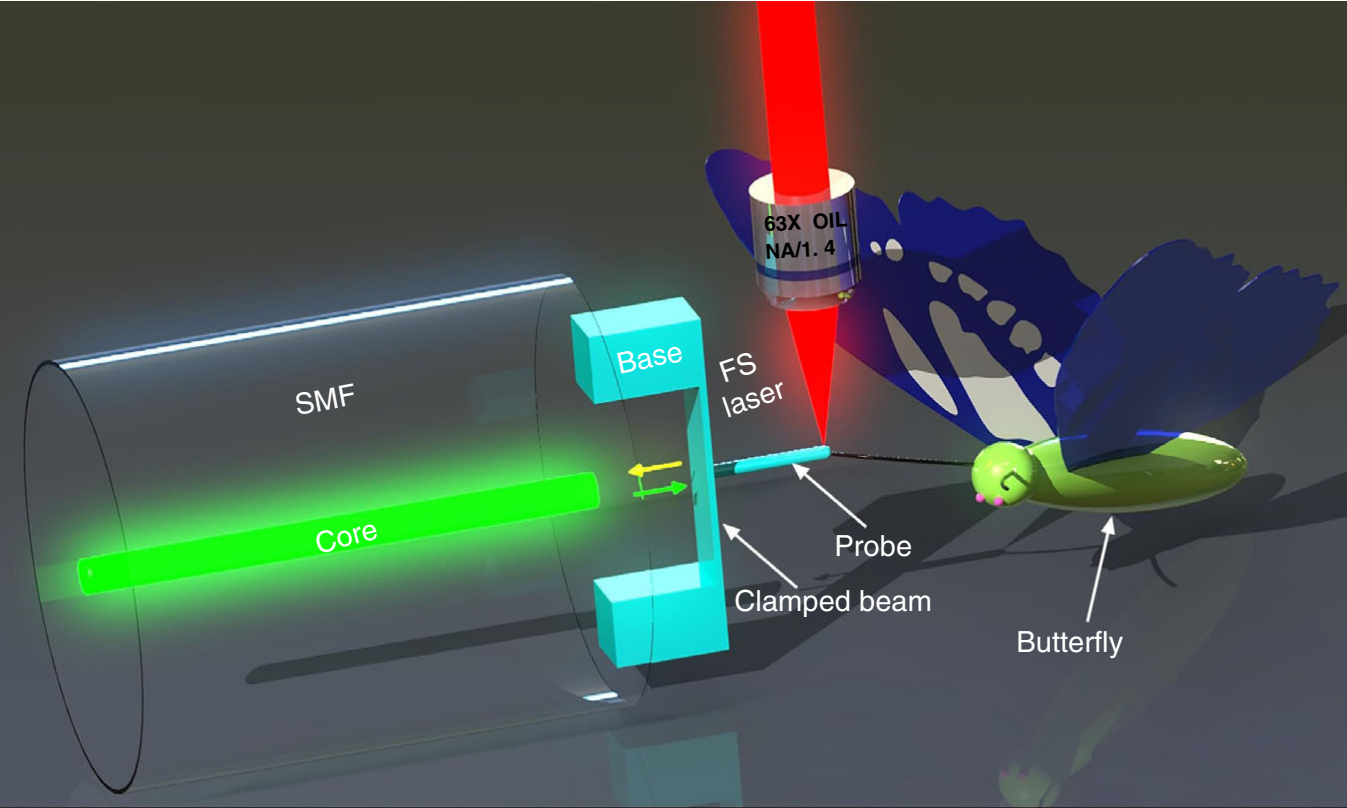
Schematic of the sensor. Schematic diagram of all-fiber micro-force sensor based on a polymer clamped-beam probe

Figure 1 presents a schematic diagram of the micro-force sensor microprinted on the single mode fiber (SMF) end face using TPP. A pair of polymer bases, with lengths of 20 μm, widths of 20 um, and heights of 30 um, were designed to support and connect the clamped beam. The clamped beam, 100 um in length, 20 um in width, and 3 um in height, was parallel to the fiber-end surface. In addition, a cylindrical probe, with a diameter of 5 um and a length of 35 um, was printed on the upper surface center of the clamped beam, and the tip of the probe was hemispherical. The lead-in fiber-end surface and the two surfaces of the clamped beam define FPIs. Firstly, the light propagating in the SMF is partially reflected at the end face of the fiber, and then the rest of the light transmitted to the lower and upper surfaces of the clamped beam will also be partially reflected into the optical fiber. These three light beams will interfere in SMF and form a reflection spectrum. Actually, three FPIs are formed, i.e., an air cavity (FPI1) formed by the fiber end face and the lower surface of the clamped beam, a polymer cavity (FPI2) formed by the two surfaces of the clamped beam, and a mixed cavity (PFI3) formed by the fiber end face and the upper surface of the clamped beam. However, in this work, the optical intensity of FPI3 is much lower than those of FPI1 and FPI2, and FPI2 has a fixed cavity length once the sensor was fabricated. Thus, the air cavity was chosen for demodulation. When the force of a small object is applied to the probe, the clamped beam will be deformed and the cavity length of FPI1 will be changed, leading to a change of dip wavelength in the reflection spectrum of SMF. The relationship between the dip-wavelength shift (Δ*λ*_*r*_) and the cavity length reduction (Δ*D*) is Δ*λ*_*r*_/*λ*_*r*_ = Δ*D*/*D*, where, *λ*_*r*_ is the dip wavelength, and *D* is the cavity length, so the force on the probe can be calculated by tracing the dip wavelength shift of the reflection spectrum. The polymer has a low Young’s modulus and stiffness^39,40^, which enables the clamped beam to deform enough under the action of small force, resulting in high sensitivity and a small force-detection limit of the sensor.

### Characterization

**Fig. 2:**
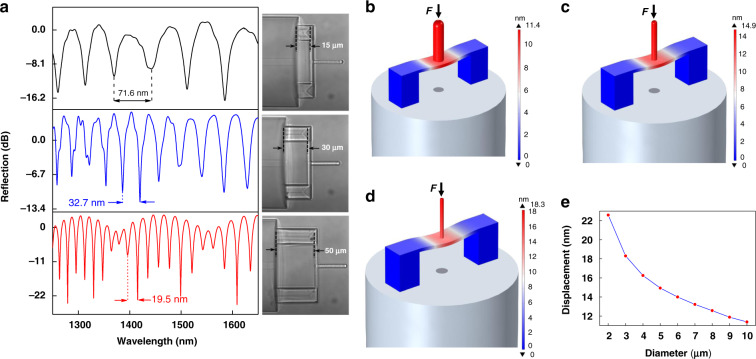
Micro-force sensor structural characterization and static performance. **a** Optical microscopy images of the clamped-beam probe with different heights and their corresponding reflection spectra. **b**–**d** are the bending deformation simulation results of the sensor under the same micro force (1 μN) acting on the probe with different diameters (10, 5, and 3 μm). **e** Relationship between the probe diameter and flexure deformation under the same micro force (1 μN)

The reflection spectra of three clamped-beam probes with different bases heights (15, 30, and 50 µm) were measured using a broadband light source (BBS, 600–1700 nm) and an optical spectrum analyzer (OSA) to optimize the structural parameters. The expression of free spectral range (FSR) is shown in Eq. 1 (ref. ^35^). The optical microscopy images of a clamped-beam probe with different heights and their corresponding reflection spectra are presented in Fig. 2a. The clamped-beam probe and the end face of the optical fiber remain parallel as the base height increases. Besides, this kind of polymer clamped-beam probe has achieved a relatively high reflection spectrum contrast (>15 dB) compared with other polymer fiber sensors based on microcantilever (<7 dB)^35^. All these characteristics show that the device microprinted by TPP has superior functions^34^. The FSRs of these three structures were measured to be 71.6, 32.7, and 19.5 nm, corresponding to the ***λ***_***r***_ of 1441.5, 1418.9, and 1415.4 nm, respectively. Combining these measured values, the heights of the bases for these three structures were calculated to be 14.5, 30.8, and 51.4 um, respectively, according to Eq. 1.

1 $${FSR} = \frac{{\lambda _r^2}}{{2nD}}$$

Where *n* is the refractive index of the medium in the cavity. These heights are consistent with the designed heights, indicating the high accuracy of TPP. In the experiment, it is found that the increase in base height can reduce the structural stability of the sensor, and it is easier to topple under the action of an external force. In contrast, a shorter base can increase the shift range of the sensor while maintaining structural stability^35^. However, if the base height is too small, i.e., the length of the air cavity is too small, the polymer structure will easily fall off when the washing solution volatilizes due to the solution surface tension between the clamped-beam and the fiber end face, reducing the development success rate of sensors. Therefore, in the sensor design, to achieve a good balance between structural stability and development success rate, we have chosen a base height of 30 μm.

To investigate the static performance of the proposed structure, models of the force sensor with different probe diameters (10, 5, and 3 μm) were established using COMSOL Multiphysics®, and the simulation results are presented in Figs. 2b, 2c, and 2d, respectively. The other values of the established simulation model were set to match the corresponding values used in the sensor-fabrication process. The measured parameters, i.e., the polymer material density of 1499 kg (m^3^)^−1^, Young’s modulus of 2.34 GPa, Poisson ratio of 0.33, and the standard parameters, i.e., silica density of 2700 kg (m^3^)^−1^, Young’s modulus of 73 GPa, and the Poisson ratio of 0.17, were employed in the simulations^41^. The same microforce of 1 μN was exerted on the probe, and the deformation distribution results are presented in Figs. 2b, 2c, and 2d, respectively. The sensor-cavity length decreases as the displacement of the probe increases. The sensor-cavity length decreased more when the probe diameter was smaller, indicating that reducing the probe diameter can effectively improve the force sensitivity of the sensor. Moreover, the relationship between the probe diameter and flexure deformation under the same microforce (1 μN) was evaluated, as shown in Fig. 2(e). The results also suggest that decreasing the probe diameter can increase the bending deformation of the sensor. The reason may be that the effective surface area of the clamped beam is the difference between the surface area of the clamped beam and the probe-lower-end area, thus the sensitivity of the sensor will be higher as the effective surface area becomes larger^11^. Therefore, a diameter of 5 μm was selected to ensure that the probe has both high mechanical strength and high sensitivity. In addition, the sensitivity of the sensor is defined as the ratio of the shift of the dip wavelength to the force, and reducing the thickness of the clamped beam can also improve its sensitivity significantly. Since the polymer material has low stiffness, the thickness of the clamped beam cannot be too small. Therefore, a thickness of 3 μm was selected to ensure the support and high sensitivity of the probe.

**Fig. 3:**
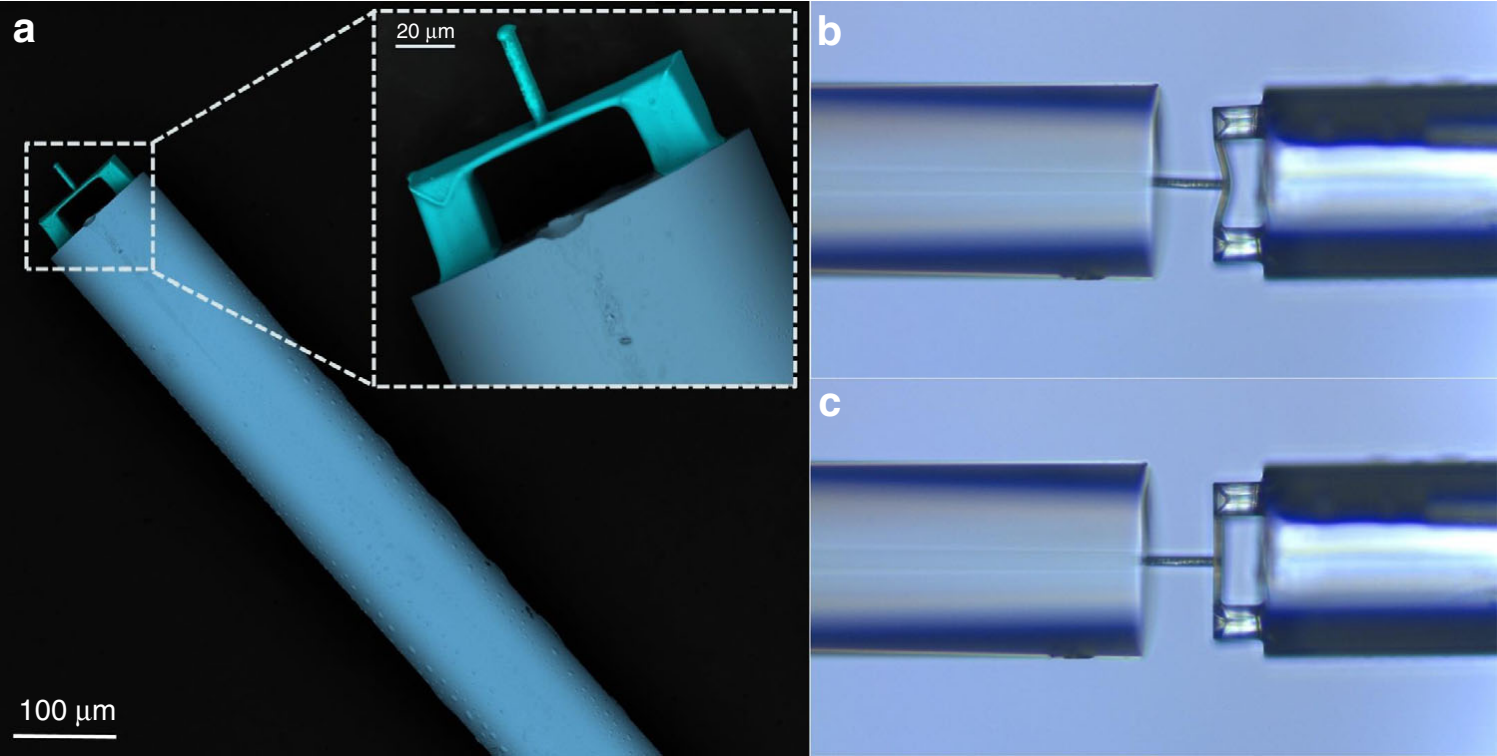
SEM images and elastic properties. **a** SEM images of fabricated polymer clamped-beam probe on the fiber tip. **b**, **c** are optical microscope images of the clamped-beam probe structure when pressed and released on the fiber-end surface, respectively

Figure 3a presents the scanning electron microscope (SEM) images of the polymer clamped-beam probe. The three main parts, i.e. the bases, clamped beam, and probe, can be clearly distinguished. The structure of the pair of bases with a cross-sectional area of 20 × 20 μm^2^ can be seen at the bottom of the figure, and a large cross-sectional area of the bases can increase the adhesion force between the polymer structure and the end face of the fiber, which makes the structure more robust. In the middle, the clamped beam attached to the bases can be seen. At the top, a cylindrical probe is located directly above the clamped beam. The SEM images show that the printed cylindrical probe has good perpendicularity with the surface of the clamped-beam, the clamped-beam has good parallelism with the end face of fiber, and the surface of the polymer structure is smooth. All these characteristics can improve both the contrast of the reflection spectrum and the sensitivity to external force, indicating the reliability of using TPP to print the microstructure on the end face of the fiber. Furthermore, the elastic properties of the prepared clamped beam probe structure were investigated by simply pressing the structure on another fiber end face. Optical microscopy images of pressing and releasing the fiber end face are presented in Figs. 3b and 3c, respectively. As observed in Fig. 3c, the probe recovered to its original state after testing.

### Force measurement

**Fig. 4:**
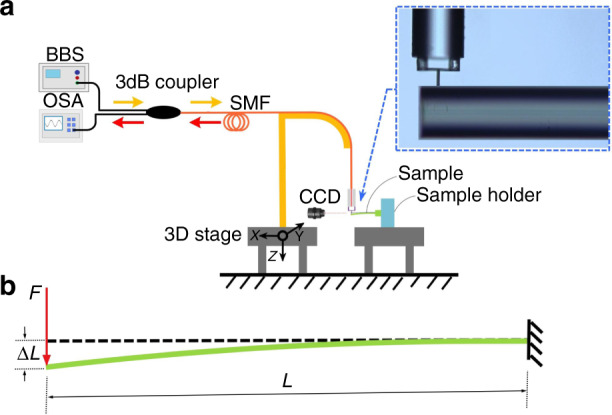
Experimental setup. **a** Measurement system setup. The inset shows a CCD image of pushing against the SMF of the clamped-beam probe sensor. **b** Schematic diagram of cantilever-beam deflection under point load

Before the microforce sensing test, the force was calibrated carefully in order to ensure measurement precision. Thus, the relationship between the applied force and the output of the sensor was quantified. The static characteristics and force sensitivity of the proposed sensor were measured using the experimental setup shown in Fig. 4a. The setup consisted of a 3D translation stage (3D stage) for micro-manipulation of the sensor, a BBS, an OSA, a 3-dB coupler, a sample holder, and a CCD camera. A section of SMF with a fixed length was mounted perpendicularly to the micro-force sensor through the sample holder. The sensor probe was pushed against the SMF to deflect it from its initial position, and the observed CCD image is shown in the inset of Fig. 4a. As observed in Fig. 4b, the diameter of the probe was much smaller than that of SMF. Thus, this process can be regarded as the deformation of a cantilever beam under a point load, which satisfies the following deflection equation (ref. ^42^):

2 $${\Delta}L = \frac{{FL^3}}{{3EI}}$$

Here, Δ*L* (mm) is the deflection of the cantilever beam. *L* (mm) is the length of the cantilever beam. *F* (kN) is the point load on the end of the cantilever beam. *E* (GPa) is Young’s modulus of the cantilever beam, and *I* is the second moment of area of the cantilever beam.

The vertical downward displacement of the probe, i.e., the deflection Δ*L*, can be accurately controlled by the 3D stage. We employed standard parameters in the calculations for SMF, including a diameter of 125 um, a length of 55.9 mm, an SMF deflection Δ*L* (obtained from the motor controller), and Young’s modulus of 73 GPa. The formula for the circular second moment of area is as follows (ref. ^43^):

3 $$I = \frac{{\pi d^4}}{{64}}$$

where *d*, in units of mm, is the diameter of the cantilever beam.

**Fig. 5:**
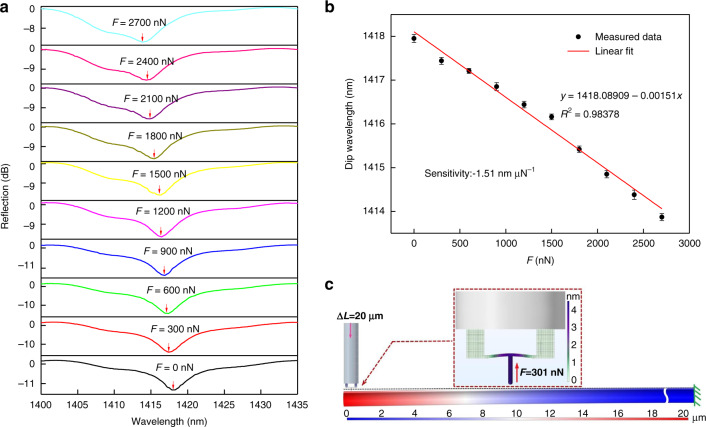
Microforce measurements and simulation. **a** Evolution of reflection spectra of the sensor as the force increased from 0 to 2700 nN, as indicated by the arrows. **b** Dip wavelength versus force. The line is the linear fitting of measured data points and the error bar is obtained by critically repeating the experiment of force measurement three times. **c** Simulation results of deformation distribution based on FEM

The forces exerted on the sensor were calculated by combining Eqs. 2 and 3. During the application of gradual force, the reflection spectrum was monitored in real time. Figure 5a shows the reflection spectral evolution of the clamped-beam probe as the force was gradually increased from 0 to 2700 nN in increments of 300 nN. A blue-shift in the dip wavelength was clearly observed, as marked by arrows. The fringe visibility of the reflection spectrum has a slight decrease as the force increases, due to the bending of the clamped-beam. The dip wavelength vs. the magnitude of the force is plotted in Fig. 5b. The dip wavelength shifted linearly toward a shorter wavelength as the applied force increases, the force sensitivity of the force sensor was calculated to be −1.51 nm μN^−1^ by using a linear fit of the dip wavelength change, which are two orders of magnitude higher than that of the previously reported fiber-optic force sensor based on a balloon-like interferometer^15^. *R*-square (*R*^2^), which describes how well the data matches the fit function, is 0.98378. It is worth noting that in our microforce sensing measurements, the clamped-beam is working within the framework of the linearly elastic range, and there is no hysteresis between the force and the cavity change. Furthermore, FEM simulation was performed; the 3D modeling of the SMF was pushed against by the clamped beam probe, and the pushing displacement was 20 um. The SMF was fixed at one end and free at the other end with a length of 55.9 mm. Figure 5c presents the simulated deformation distribution result. When a 20-um displacement occurred at the free end of the SMF, the bending deformation of the clamped beam and the cavity length decreased due to the action of reaction (in the insets of Fig. 5c), and the reaction force was 301 nN, which is well consistent with the experimental increment step. In addition, the external force not perpendicular in the practical application can also be measured (see Fig. S3 in supporting information). The force sensitivity of the proposed sensor can be further improved by reducing the polymerization thickness of the clamped-beam when the stiffness is allowed, for the force sensitivity of the sensor is inversely proportional to the third power of the thickness of the clamped-beam^44^. Although the robustness and mechanical durability to other loads, such as lateral forces, were not tested systematically, none of the sensors failed in a wide range of experiments in this study. This might be attributed to the excellent surface quality (obtained by TPP processes) and the inherent high elasticity of polymer structure^45^. The experimental results show that the maximum force measurement of the sensor is 2.9 mN.

I. M. White et al. introduced the concept of sensor detection limit (*DL*_s_), in which the spectral resolution and correlated noise parameters of the system were fully considered^46^. Herein, the detection limit of the micro-force sensor is the definition of the smallest applied-force variation that can be accurately measured. The *DL*_s_ is expressed as

4 $${DL}_{{{\mathrm{s}}}} = \frac{{R_s}}{{S_s}}$$

where, *S*_s_ and *R*_s_ represent the sensitivity and resolution of the sensor, respectively, and the resolution of the sensor can be approximately solved by individual noise variances (namely, *R*_s_ = 3*σ*), i.e.,

5 $$\sigma \approx \frac{{{\Delta}\lambda _{{{\mathrm{F}}}}}}{{4.5({SNR}^{0.25})}}$$

Here, Δ*λ*_F_ is the full-width at half-maximum (FWHM) of the fringe. *SNR* is the signal-noise ratio.

In our micro-force sensing experiment, the main restriction of the detection limit is the FWHM. The measured value of FWHM is 2.21 nm, and the *SNR* expressed in linear units is 50 dB. The *DL*_s_ of the device was calculated to be 54.9 nN. Such an ultra-small *DL*_s_ value can help the proposed sensor detect tiny force variations. With its ability to measure forces from the nN to mN level, this probe sensor has a force measurement range spanning of more than five orders of magnitude, which is expected to facilitate cross-scale force sensing.

Table 1 compares the performance of optical-fiber sensors with different configurations for micro-force measurement. In terms of force sensitivity, the proposed micro-force sensor is much higher than other fiber-optical force sensors types. In addition, the device has the advantages of flexible fabrication, high mechanical strength, an ultra-small detection limit, and ultra-compactness.

**Table 1:** Comparison of performance of fiber sensors with different configurations for micro-force measurements

Sensor structure	Size of sensor	Force sensitivity (pm μN^-1^)	Reference
Photonic crystal fiber	125 μm × 3 cm	0.016 × 10^−3^	^54^
Microfiber Bragg grating	2.4 mm × 2.4 mm	0.73 × 10^−3^	^55^
FP cuboid cavity	18 μm × 60 μm	0.026	^56^
Microfiber asymmetrical FP interferometer	20 mm × 7.3 um	0.221	^57^
FP micro-cavity plugged by cantilever taper	1.36 mm × 125 μm	0.842	^58^
Balloon-like interferometer	24 mm × 14 μm	24.9	^15^
Clamped beam probe	68 μm × 100 μm	1510	This work

### Sensing applications

**Fig. 6:**
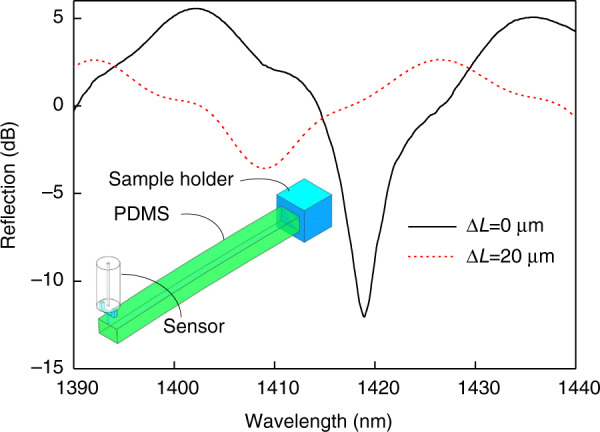
Evolution of reflection spectrum of the sensor as PDMS deflects from 0 to 20 μm. As shown in the inset, one end of the PDMS sample is fixed while the other end is deflected by the proposed sensor

The proposed sensor was applied in a few typical applications, including the measurement of Young’s modulus of PDMS, a butterfly feeler, and human hair. To obtain the required PDMS sample, the elastomer and cross-linking agent were mixed with a ratio of 10:1, degassed for 30 min, and then cured in an oven at 100 °C for 60 min. The PDMS sample was first cut into pieces with a size of 17.7 mm × 1.27 mm and a thickness of 1.03 mm. Then, one end was fixed into the sample holder and the other end was deflected by a 3D stage with an attached micro-force sensor, as shown in the inset in Fig. 6. Figure 6 presents the reflection spectra of the sensor when the PDMS was in the initial state (Δ*L* = 0 μm) and deflected by 20 μm. When the PDMS was pushed down with a distance of 20 μm, the reflection spectrum blue-shifted, and the dip wavelength was 1409.03 nm. According to the linear fitting function between dip wavelength and force applied, the tested force was calculated to be 5999.397 nN. However, the calculation for the rectangular second moment of area differs from that of the circular second moment (ref. ^43^):

6 $$I = \frac{{bh^3}}{{12}}$$

Here, *b* (mm) is the width of the cantilever beam and *h* (mm) is the thickness of the cantilever beam. Thus, Young’s modulus of PDMS was calculated to be 4.8 MPa by combining Eqs. 2 and 6, using the geometry of PDMS, i.e., *L* = 17.7 mm, *b* = 1.27 mm, *h* = 1.03 mm, and point load *F* = 5999.397 nN on the end of the PDMS. Our value (4.8 MPa) of Young’s modulus are comparable to those obtained on the PDMS in both Seghir et al. that ranged from 0.8 to 10 MPa^47^ and Chaudhury et al. that ranged from 0.2 to 9.4 MPa^48^, which certifies the reliability of the proposed sensor.

To verify the calculated results of Young’s modulus of PDMS, an AFM (Bruker) was used to perform mechanical measurements on the same PDMS sample. In AFM measurement, a 20 × 20 μm area of the PDMS sample is selected for depth-sensing indentation experiment, and the total length of the vertical ramp is kept at 5 μm. We used a standard sharp indenter model RTESPA with an average semi-angle aperture *θ* = 20° (measured by SEM). The elastic spring constant, i.e., *k* = 18.7 Nm^−1^ was calibrated in the air using the thermal tune method^49^. The AFM tip was indented vertically on the surface of PDMS to obtain the force indentation curve, so that the morphology and mechanical properties of the samples were obtained. In order to improve the reliability of experimental data statistics, the indentation experiments were repeated three times at room temperature (*T* = 23 °C).

The Young’s modulus of PDMS was obtained by fitting the force indentation curve obtained from AFM depth-sensing indentation experiment in Matlab^50^. According to the shape of the AFM probe used, the Sneddon model is used to fit, and the expression is as follows^51,52^.

7 $$F = 0.7453\frac{{E\tan \theta }}{{\left( {1 - \nu ^2} \right)}}\delta ^2$$

Here, *E* represents the local Young’s modulus of the sample, *ν* is the Poisson ratio, and *θ* represents the average half-opening angle of the AFM probe.

**Fig. 7:**
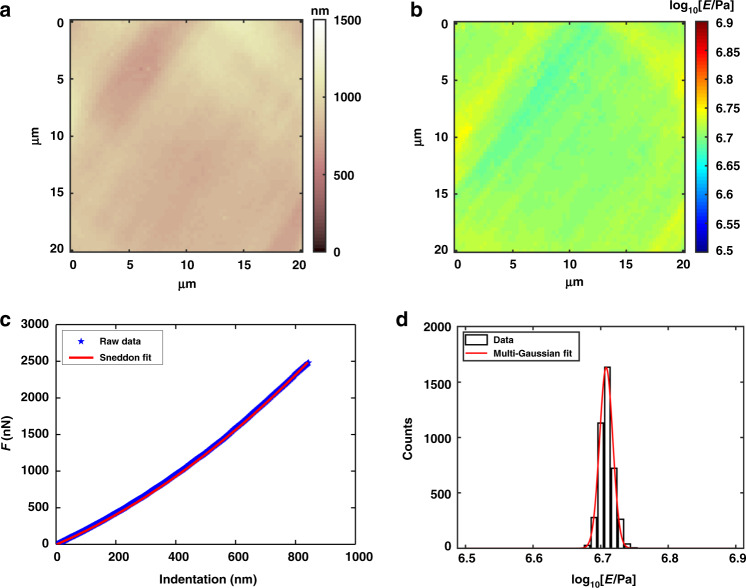
Graphical results of mechanical analysis by AFM. **a** Force volume morphology map of PDMS thin film, the color bar indicates height value. **b** Young’s modulus mechanical diagram on a logarithmic scale. **c** The Young’s modulus of the sample was estimated by Sneddon model fitting based on the force indentation experimental data. **d** Histograms of Young’s modulus values, the red curve is Gaussian fitting

The AFM results measured are shown in Fig. 7. Figure 7a shows the morphology of the PDMS obtained by AFM. The surface topography of the PDMS film was uniform, and the root mean square roughness was determined to be 87.4 nm. Figure 7b presents Young’s modulus mechanical map of the PDMS thin film. The vertical color bar is in the form of logarithmic coordinates. The Young’s modulus distribution of PDMS was relatively uniform, with an average level of 5.11 ± 0.01 MPa. Force indentation curves of the sample are presented in Fig. 7c. The black dotted line represents the measurement data, and the red line represents the Sneddon fit curve. The histogram of Young’s modulus is shown in Fig. 7d and the red curve is Gaussian fitting. The Young’s modulus of PDMS was mainly concentrated around 5.11 MPa. The measurement results of the proposed micro-force sensor are mostly consistent with the AFM results, and the error is within 10%, which certifies the accuracy of the measured results of the proposed sensor.

**Fig. 8:**
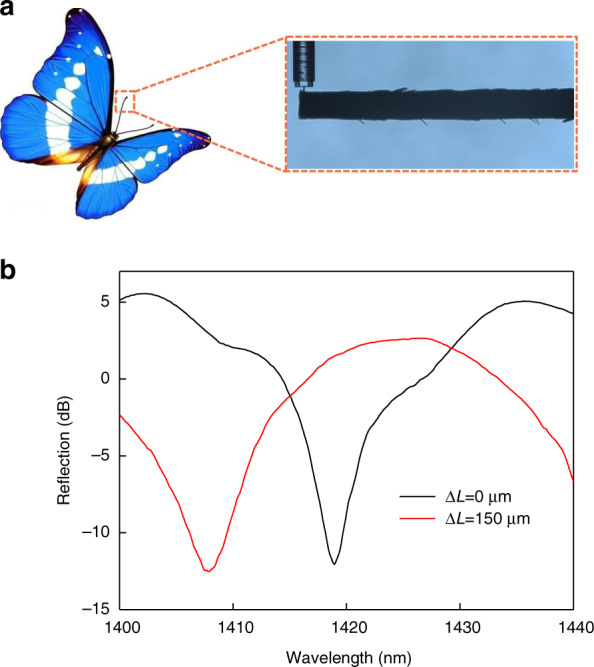
Butterfly feeler measurement. **a** CCD image of pushing against butterfly feeler of the proposed sensor. **b** Evolution of reflection spectrum of the sensor with deflection of butterfly feeler from 0 to 150 μm

Similarly, one feeler of a butterfly (Danaidae) was fixed into the sample holder, similar to the PDMS measurement process. The free end of the butterfly feeler was pushed by the micro-force sensor probe, as shown in Fig. 8a, thus the feeler was deflected from its initial position. Figure 8b presents the reflection spectrum of the sensor when the butterfly feeler was in the initial state (Δ*L* = 0 um) and when it was deflected 150 um from the initial state. When the butterfly feeler was pushed down at a distance of 150 μm, the dip wavelength became 1407.87 nm. The Young’s modulus of the butterfly feeler can be calculated according to the asserted forces measured by the sensor and the geometric (butterfly feeler, *L* = 8.2 mm, *d* = 165 μm), similar to the aforementioned calibration process. The Young’s modulus was calculated to be 227.8 MPa for the butterfly feeler and ~5.65 GPa for the human hair (black hair from Chinese adult women), which agrees well with the findings reported in biological literature^53^.

## Discussion

In this study, we demonstrated a miniature all-fiber micro-force sensor, in which the clamped beam parallel to the fiber end face, the support bases, and the force-sensing probe were printed on the SMF end surface using the TPP 3D microprinting method. A linear force response was achieved with a high sensitivity of 1.51 nm μN^−1^ and a force detection limit of ~54.9 nN. In addition, the measured maximum value of the sensor can be 2.9 mN. The proposed sensor can be used to measure the mechanical properties (e.g., Young’s modulus) of elastoplastic materials and biological samples. Our static characteristic simulations provided validation of our experimental results as well as further insight. With its micrometer scale, absence of special packaging requirements, all-dielectric design, biocompatibility, and all-fiber operation, the proposed sensor has great application prospects for investigation of the mechanical properties of materials and examination of biological samples. In the future, we expect that the novel optical-fiber sensor can be widely used in long-distance biological detections such as biological-cell detection and in vivo elastography of tissues.

## Materials and methods

**Fig. 9:**
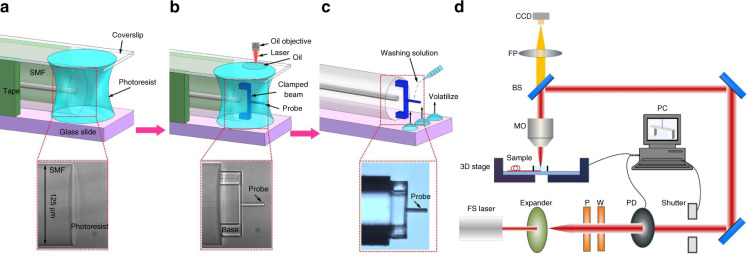
Fabrication process of the device. **a** A drop of photoresist solution is dropped into the end face of the SMF, and the coverslip is pressed on the side of the fiber to prevent the photoresist from flowing during polymerization. **b** Femtosecond laser polymerizes a clamped-beam probe structure on the end face of the fiber **c** Remove the coverslip on the top of the optical fiber and wash the uncured photoresist with the cleaning solution composed of acetone and isopropanol. **d** Schematic diagram of the optical path of the TPP micro–nano processing

Figure 9 demonstrates the fabrication process of femtosecond laser TPP microprinting fiber end clamped-beam probe, which mainly involves three steps.

Figure 9a shows the first step of the fabrication process. Firstly, a standard SMF (Corning SMF-28) is placed on a glass slide, before being placed on the glass slide, the fiber end is carefully cleaned and well cleaved. Three layers of adhesive tape are pasted as spacers on the glass slide on both sides of the optical fiber end. Then, a drop of photoresist solution is dropped onto the end face of the SMF, thus the fiber end of the SMF is covered with the photoresist solution. To prevent the photoresist from flowing during the polymerization, one coverslip is pressed on the upper surface of the optical fiber. The photoresist used in this study contains a photo-initiator (IGR-369) and monomer (SR444, SR369). A microscope image of the fiber tip surrounded by photoresist solution is shown at the bottom of Fig. 9a.

Figure 9b shows the second step of the fabrication process. The sample with a photoresist on the fiber tip is fixed on the 3D displacement stage through air pressure. Two drops of refractive-index-matching solution are added to the coverslip, and the oil immersion objective of 63×magnification (NA = 1.40) is used for it can help make the linewidth of the structure as small as possible, and improve the polymerization resolution. The femtosecond laser, with a central wavelength of 1026 nm, is focused on the fiber tip through both the objective lens and the coverslip. Import the 3D image designed to the control software (SCA lntro v2.6), and control the laser, the clamped-beam probe structure is polymerized according to the predesigned scanning path. Note that the quality of polymer structure will be affected by the femtosecond laser power and the scanning speed. The power is set to be 1.6 mW and the scanning speed is 0.5 mm s^−1^ after optimization. A microscope image of the clamped-beam probe structure polymerized on the end face of the optical fiber is shown at the bottom of Fig. 9b.

Figure 9c shows the third step of the fabrication process. Remove the coverslip on the top of the optical fiber carefully and wash out the uncured photoresist with the cleaning solution. Acetone and isopropanol are mixed to prepare the cleaning solution according to the ratio of 1:5. Added two drops of the mixture cleaning solution carefully to the end face of the optical fiber, and repeat it after the cleaning solution volatilizes. After washing three times, the uncured photoresist is clean, repeat it if necessary. Then, the micro-force sensor of the all-fiber clamped-beam probe is obtained, as shown in the lower part of Fig. 9c.

A schematic diagram of the optical path of the TPP micro-nano processing is presented in Fig. 9d. The Fs laser beam first passed through a beam expander, a variable attenuator composed of a λ/2 wave plate (W), a Glan prism polarizer (P), an optical power dynamometer (PD), a shutter, and a series of mirrors, and then the output laser was focused on the photoresist on the fiber tip through an oil immersion objective. The sample was mounted on a 3-Axis translational stage (Aerotech, X, Y-axis: ABL1500, Z-axis: ANT130V-5) with a spatial accuracy of ±200 nm and a resolution of 2 nm. The NPAQ servo controller (Aerotech Inc.), controlled by the software (SCA lntro v2.6), was used to adjust the motion path and velocity of the 3-Axis translational stage. Thus, by keeping the focal point still, and preciously controlling the relative position between the focal point of the objective and the sample to be processed through the software, then the fabrication of an arbitrary polymer 3D structure could be realized. The process of femtosecond laser TPP microprinting clamped-beam probe can be monitored by a CCD camera. The process of femtosecond laser TPP microprinting clamped-beam probe was monitored by a CCD camera. The whole printing process was observed in real time to monitor whether the laser power set, the scanning speed, and other processing parameters are within an appropriate range. Simultaneously, the structural integrity of the clamped-beam probe is observed, thus improving the processing quality of the sample.

## Supplementary information

Supplementary Information

## Data Availability

The data that support the findings of this study are available from the corresponding author upon reasonable request.
