# Supplementary material for: Fiber-tip polymer clamped-beam probe for high-sensitivity nanoforce measurements
Source: Light Sci Appl. 2021 Aug 27;10:171. doi: 10.1038/s41377-021-00611-9 (PMC8397746; doi:10.1038/s41377-021-00611-9)
Supplement: Supplementary file 1 — Supplementary Information [file 41377_2021_611_MOESM1_ESM.pdf]

Supplementary Information

Fiber-tip polymer clamped-beam probe for high-sensitivity nanoforce measurements

Mengqiang Zou[1,2], Changrui Liao[1,2,*], Shen Liu[1,2], Cong Xiong[1,2], Cong Zhao[1,2], Jinlai Zhao[3], Zongsong Gan[4,5], Yanping Chen[1,2], Kaiming Yang[1,2], Dan Liu[1,2] ,Ying Wang[1,2] and Yiping Wang[1,2,*]

[*]*Correspondence: Changrui Liao (cliao@szu.edu.cn) or Yiping Wang (ypwang@szu.edu.cn)*

[1]*Key Laboratory of Optoelectronic Devices and Systems of Ministry of Education/GuangDong Province, College of Physics and Optoelectronic Engineering, Shenzhen University, Shenzhen 518060, China*

[2]*Shenzhen Key Laboratory of Photonic Devices and Sensing Systems for Internet of Things, Guangdong and Hong Kong Joint Research Centre for Optical Fibre Sensors, Shenzhen University, Shenzhen 518060, China*

[3]*College of Materials Science and Engineering, Shenzhen Key Laboratory of Polymer Science and Technology, Guangdong Research Center for Interfacial Engineering of Functional Materials, Shenzhen 518060, China*

[4]*Wuhan National Laboratory for Optoelectronics (WNLO), Huazhong University of Science and Technology (HUST), Wuhan 430074, China*

[5]*Shenzhen Huazhong University of Science and Technology Research Institute, Shenzhen, Guangdong, 518057, China*

Section 1-Temperature Measurement

To study the temperature cross interference of the sensor for a general biomedical environment, the effect of temperature was investigated. The sensor was placed into an oven in air and we gradually increased the temperature from 25℃ to 55℃ with a step of 5℃. The temperature was maintained for 15 min at each step. The evolution of the reflection spectrum as the temperature increases is shown in Fig. S1a. The dip wavelength at ~1437 nm was observed to be a red shift with increasing temperatures. The dip wavelength shift of the FPI spectrum with temperature can be expressed by the following equation,

$$\frac{\mathrm{d}\lambda}{\mathrm{d}T}=\frac{2}{k}\left(\frac{\mathrm{d}n}{\mathrm{d}T}L+\frac{\mathrm{d}L}{\mathrm{d}T}n\right) \tag{S1}$$

Where λ and k are the dip wavelength and an integer representing the order of the interference spectrum, respectively. $\frac{dn}{dT}$ and $\frac{dL}{dT}$ are the thermo-optic coefficient of the medium in the cavity and the thermo-expansion coefficient of the polymer, respectively. Both thermo-optical effect and thermal expansion effect contribute to the shift of interference spectrum when the ambient temperature rises. However, since the medium in the air cavity formed by the fiber end face and the lower surface of the clamped-beam is air, the refractive index of air barely changes as the temperature increases within the range of 25℃ to 55℃[1]. Therefore, we believe the dominant reason is that the thermal expansion of the polymer bases of the clamped-beam probe changes, leading to an increment of the cavity length. Figure S1(b) illustrates the linear fit of the dip wavelength versus temperature, achieving a sensitivity of ~47.36 pm/℃, which is consistent with the temperature sensitive characteristics of the previously reported photonic crystal fiber force sensor (45 pm/℃)[2]. The R^2 of the linear fit is calculated to be 0.997 with a standard error of 1.08 pm. Thus, this polymer clamped-beam probe has achieved a temperature cross-sensitivity of ~ -0.031 μN/℃. Moreover, our microforce sensing experiments are carried out in an ultra-clean room with a constant temperature and humidity, and the temperature fluctuation is less than ±0.1 ℃ in the force measurement period, so the effects of the temperature fluctuation to the sensor can be ignored in this work.

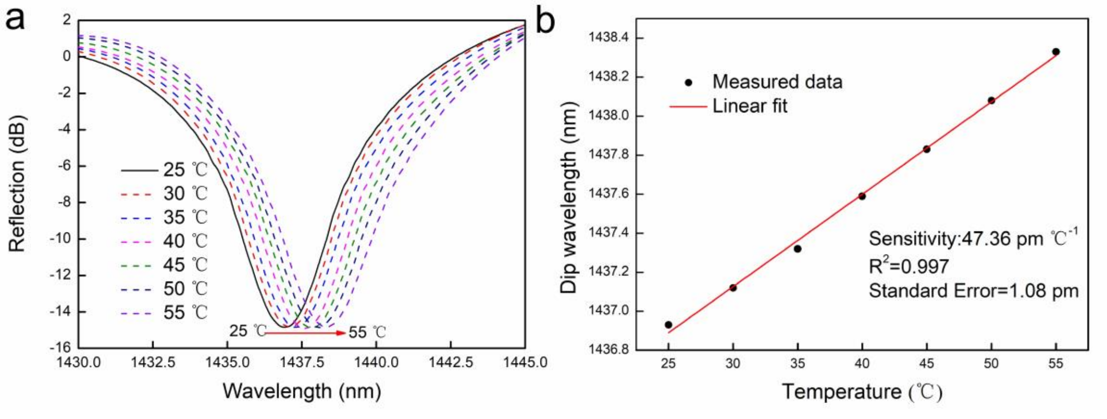

Fig. S1. Temperature response of the polymer clamped-beam probe. a Reflection spectrum evolution of the polymer clamped-beam probe while the temperature increases from 25 to 55℃. b Data and a linear fit of the dip wavelength versus temperature.

Section 2-Humidity Measurement

In order to study the influence of humidity on the sensor in atmospheric environment, the humidity response of the sensor was measured in a constant humidity chamber within the range of 30% ~ 90%. A 15-minute stabilization is performed at each measurement step to ensure that the sensor is fully responded. Figure S2(a) shows the reflection spectrum evolution of the sensor as the humidity increases from 30% to 90% with a step of 10%. Obviously, the spectrum has a significant red shift as

the ambient humidity increases. The dip wavelength shift with humidity can be expressed by this equation

$$\frac{d\lambda}{d\varphi} = \frac{2}{k}\left(\frac{dn}{d\varphi}L + \frac{dL}{d\varphi}n\right) \tag{S2}$$

where λ is the dip wavelength, k is an integer representing the order of the interference spectrum, $\frac{dn}{d\varphi}$ is the change of refractive index with humidity in FP cavity and $\frac{dL}{d\varphi}$ is the hygroscopic-expansion coefficient of the polymer. The red shift of reflection spectrum can be attributed to both the increase of FP cavity length caused by the hygroscopic expansion[3] of polymer support bases and hygroscopic deformation of clamped beam and the refractive index of air changes with humidity[4]. The humidity sensitivity is calculated to be 118 pm/% with a standard error of 4.45 pm by a linear fit to the dip wavelength measured, as shown in Fig. S2(b). A humidity cross- sensitivity of -0.078 μN/% for the sensor is obtained, which is of great significance to minimize the influence of humidity on the performance of sensors in biosensor applications.

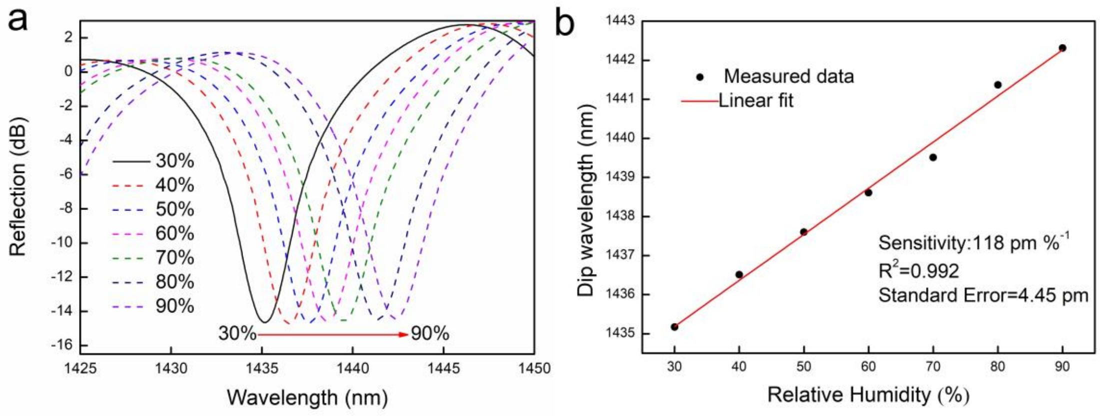

Fig. S2. Humidity response of the polymer clamped-beam probe, indicates a linear relationship between the dip wavelength and the relative humidity. (a) Reflection spectrum red shift as the relative humidity increased from 30% to 90%. (b) Data and a linear fit of the dip wavelength versus relative humidity

Section 3- Tilting force Measurement

As shown in Fig. S3, when the force applied to the probe is not perpendicular to the surface of the clamped-beam, and assuming the angle between the force F and the surface of the clamped-beam is α, the force **F** applied can be decomposed into the force F1 perpendicular to the surface of the clamped-beam through $F1 = F\sin(\alpha)$ and the force F2 parallel to the surface of the clamped-beam by $F2 = F\cos(\alpha)$. The force F2 is a lateral force, which makes the probe bend laterally, and the lateral force will be transferred to the bases of the clamped-beam probe, thus affecting the firmness of the adhesion between the bases and the surface of the optical fiber. If F2 is enough, the probe will break and/or the polymer structure will fall from the surface of the optical fiber. The force F1 will cause bending deformation of the clamped-beam, resulting in an FPI cavity length change, that is, F1 is the actual force detected by the sensor. Therefore, there will be a certain deviation between the actual applied force F and the detected force F1. In the experiment, in order to deal with the situation that the external force is not perpendicular in the practical application, we can measure the angle α between the applied force F and the surface of the clamped-beam, combine the force F1 detected by the sensor, and obtain the applied force F through the equation $F = F1/\sin(\alpha)$. Thus, the force of the non-perpendicular probe can be obtained without reducing the measurement accuracy of the sensor.

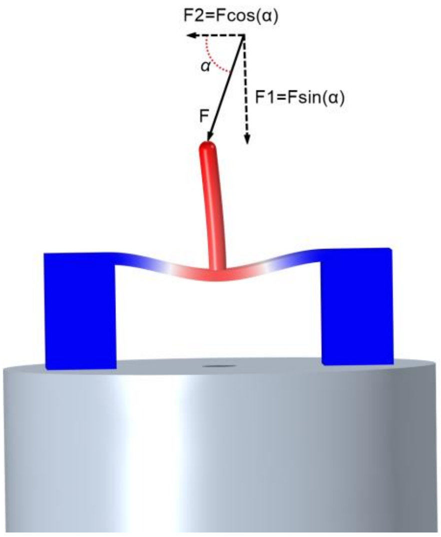

Fig. S3. Simulation result of bending deformation of clamped-beam probe under non perpendicular force

Section 4- Finesse Improvement

The polymer material employed has a refractive index of ~1.53, which is the same as that in our previous work in Ref. 5. Then, the reflectivity R=~4.439% of the polymer material is obtained by calculating through $R = \frac{(n_1 - n_2)^2}{(n_1 + n_2)^2}$, where n_1 and n_2 are the refractive indices of the air and the polymer material, respectively. Yes, it is possible to deposit reflection coatings, for example, Au, on the surfaces of the fiber tip and the polymer material after the clamped-beam is made to increase the Finesse of the FPI. Theoretically, the finesse[6] F of FPIs is calculated through $F = \pi (R_1 R_2)^{1/4} / (1 - \sqrt{R_1 R_2})$, where R_1 and R_2 are the reflectivity of the surface of the fiber tip and the lower surface of the clamped beam, thus an increase of the reflectivity R leads to a larger finesse F. Depositing reflection coatings on the surfaces of the fiber tip and the polymer material after the clamped-beam can increase the reflectivity of these two surfaces, thus increasing the finesse of the FPI. The corresponding reflection spectrum before and after depositing Au are shown in the attached figure below (Fig. S4). Yes, a high Finesse can improve the performance of the sensor. An FPI with a high finesse will have a reflection spectrum with sharp dips. Sharp dips can help measure the resonance dip wavelength movement more accurately, thus improving the measurement accuracy of the sensor. Besides, higher finesse helps improve the detecting limit of the sensor. Taking high finesse into account is a very good suggestion, which will be performed in the next work.

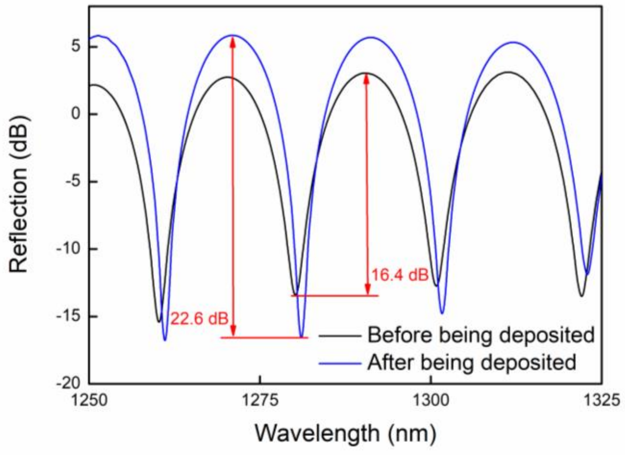

Fig. S4. The corresponding reflection spectrum before and after depositing Au

Reference

1. Xiong, C. et al. Fiber-Tip Polymer Microcantilever for Fast and Highly Sensitive Hydrogen Measurement. ACS Appl Mater Interfaces **12**, 33163-33172 (2020).
2. Liu, Q. et al. High-sensitivity photonic crystal fiber force sensor based on Sagnac interferometer for weighing. Optics & Laser Technology **123**, 105939 (2019).
3. Schmid, S., Kuehne, S. & Hierold, C. J. Influence of air humidity on polymeric microresonators. *J. Micromech. Microeng* **19**, 065018 (2009).
4. Wesely, M. L. The Combined Effect of Temperature and Humidity Fluctuations on Refractive Index. *J. Appl. Meteorol* **15**, 43-49 (1976).
5. Li, C. et al. Femtosecond laser microprinting of a polymer fiber Bragg grating for high-sensitivity temperature measurements. Opt. Lett. **43**, 3409-3412 (2018).
6. Lawrence, M. J. *et al.* Dynamic response of a Fabry–Perot interferometer. *J. Opt. Soc. Am. B* **16**, 523-532 (1999).
